# Quantifying Hole-Edge Crack of Bolt Joints by Using an Embedding Triangle Eddy Current Sensing Film

**DOI:** 10.3390/s21072567

**Published:** 2021-04-06

**Authors:** Shilei Fan, Junyan Yi, Hu Sun, Fenglin Yun

**Affiliations:** 1 College of Aerospace Engineering, Nanjing University of Aeronautics & Astronautics, Nanjing 210016, China; fanshilei@comac.cc; 2 COMAC Shanghai Aircraft Design & Research Institute, Shanghai 201210, China; 3 School of Aerospace Engineering, Xiamen University, Xiamen 361005, China; junyanyi@stu.xmu.edu.cn (J.Y.); yunfl@xmu.edu.cn (F.Y.)

**Keywords:** structural health monitoring, crack quantification, triangle eddy current coils, sensing film

## Abstract

Hole-edge crack quantification of bolt joints is critical for monitoring and estimating structural integrity of aircraft. The paper proposes a new triangle eddy current sensor array for the purpose of increasing the level of quantifying hole-edge crack parameters, especially, the crack angle. The new senor array consists of triangular coils instead of planar rectangular coils. The configuration of the novel sensor array, including the excitation current directions and the excitation winding shape, is optimized by simulation. The ability of the proposed sensing film to identify the crack parameters has been verified by finite element simulations and experiments. Results shows that triangular coils with same current directions in circumferentially adjacent coils and opposite current directions in axially adjacent coils achieve better performance in sensor linearity and resolution compared to rectangular coils. In addition, it has also been proved that the sensing film has a good potential to identify the crack depth and length.

## 1. Introduction

Bolt joints are the most important connection types of key structures for aircraft, meanwhile, they are also the weak link due to the stress concentration caused by the opening bolt hole. Eighty percent of structural mechanical failures are caused by failure of jointed structures [1]. It is critical to monitor and prevent the damage and failure of bolt joints around the hole to ensure the structural integrity and the operational safety of advanced equipment.

In addition to the bolt-loosening failure, there are three typical hole-edge failure modes: shear failure, tensile failure and bearing failure [2,3]. According to the characteristics of structural force, the crack distribution of three failures along the circumferential angle of the bolt hole is obviously different (shear failure [30°, 60°], tensile failure [75°, 105°] and bearing failure [−15°, 15°]), which can be used to distinguish failure modes by crack angle.

In the service process of aircraft, if the damage initiation and growth in the early stage of bolt joint structure failure is monitored online and real-time by sensor network integrated with the structure, it will be helpful to monitor the failure evolution process of bolt joints and predict the hole-edge failure mode of the jointed structure, and provide technical and data support for optimizing the design of bolted joints. Structural health monitoring (SHM) technology can conduct real-time quantitative monitoring of the structure state and possible damage by using the sensor network integrated with the structure. SHM is a revolutionary innovation technology to determine the structural integrity and a key technology to improve the operation safety of advanced equipment, and moreover can improve the present design concept of damage tolerance [4]. There are some SHM technologies which have been used for the damage monitoring of bolted joints, such as ultrasonic guided wave [5,6,7], electromechanical impedance [8,9], comparative vacuum monitoring sensor [10], vibration [11], acoustic emission [12] and so on. However, these technologies have the following problems: (1) It is difficult to identify the cracks in the non-surface layer of a multi-layer jointed structure; (2) It is difficult to accurately quantify the angle of hole edge cracks, and then identify the failure mode.

Eddy current (EC), which is low cost and efficient for dealing with conductive bodies, has been the focus of many researchers. There are three main types of flexible EC array sensor films developed for monitoring the crack growth around the bolt hole. The first is meandering winding magnetometer arrays (MWM) proposed by JENTEK Sensors Inc. [13], which is actually designed specifically to measure the electrical conductivity and permeability of the material first and is further applied by Goldfine et al. [13,14,15,16,17] to detect fatigue cracks on metal structures. It is found that MWM is able to track crack growth with a resolution of 50 um. However, the output signal of the MWM is too small to be detected. The second one is the rosette-like eddy current array (RECA) sensor, which is composed of circular drive wingdings periodically deposited on the flexible film and pickup winding array distributed between the adjacent drive windings. Li et al. [18] enhanced the sensitivity of the sensor by placing a magnetic membrane washer on the surface-mounted sensor film. As found in [19], mutual interference between the channels affects the amplitude and phase of the transimpedance. Chen et al. [20] proposed a new layout of the pickup coils. Sensing channels in the outer layer and in the inner layer are misaligned to improve the angular resolution of the RECA sensor. They also discovered that exciting currents in adjacent wingdings flowing in same directions instead of opposite directions can increase the output signal and sensitivity. Fan et al. [21] further studied the effect of the excitation layout on the sensor sensitivity and the results show that the sensitivity was increased up to 43.1 times in simulation and 23.7 times in an experiment when the co-directional excitation coil layout was applied. Furthermore, research projects into the temperature compensation for RECA sensor have also been conducted in [22,23]. The RECA sensors perform well in tracking crack propagation and crack angle identifying. Nevertheless, it is hard for them to discover cracks deep in the plate due to the limit of the skin effect which is unavoidable when applying eddy current.

**Figure 1:**
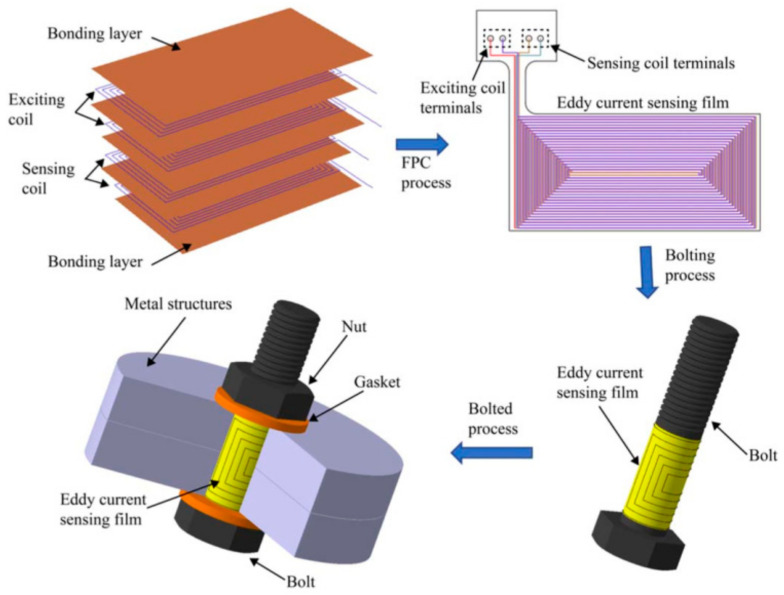
An eddy current (EC) sensing film-based intelligent bolt.

The third type of flexible EC array sensor is the intelligent bolt with flexible sensing film integrated on the surface of the bolt, which can detect the crack deep in thick plates or multi-layer jointed structure, as shown in Figure 1. Rakow and Chang [24] firstly proposed the concept of eddy current-based intelligent bolt and studied the capacity of the film to track flaw depth. Sun et al. [25,26,27] optimized the coil layout in order to offer more accurate information about the crack. First, an EC sensing array uniformly distributed along the axial direction was proposed to monitor the crack depth [25]. Furthermore, a 2D EC array-based sensing film [26] consisting of a 3 × 3 coil array was developed to identify the failure mode by providing the crack positions on the joint structure. However, although this proved to have the ability to identify the cracks in different sensing regions, planar rectangular coils are unable to provide a precise crack angle for the fact that there are blind zones existing in the interface between two adjacent sensing coils. Thus, this paper presents a new sensing array which is composed of triangular coils, and it also provides inspiration for sensor design in other areas.

The rest of the paper is organized as follows: the capability of the proposed sensing array to identify crack angle is demonstrated by the combination of the simulation in Section 2 where the triangular exciting coils and the rectangular exciting coils are compared and the experiment in Section 3; conclusions are drawn in Section 4.

## 2. Design and Analysis of 2D-Triangular Eddy Current (EC) Sensing Film

**Figure 2:**
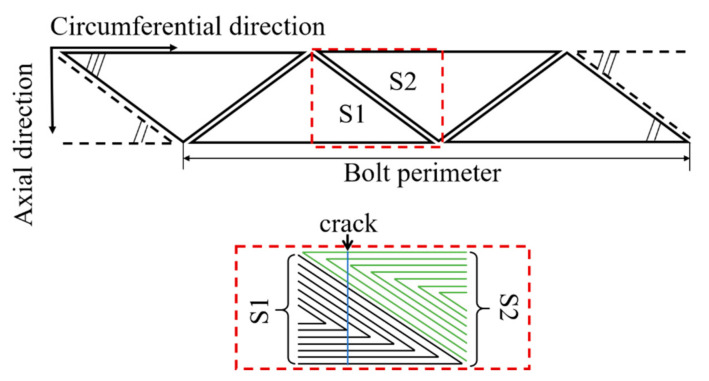
The simplified triangular sensing coil array configuration.

For the purpose of improving the hole-edge crack angle identification ability, a novel 2D EC sensing array film that consists of triangular sensing coils instead of rectangular sensing coils used in [25] is proposed in this paper, and a distribution schematic of its pickup coil array is presented in Figure 2. As is shown in the picture, there are 4 triangular pickup coils in the circumferential direction and each coil covers half of the bolt hole perimeter. As depicted in the red dashed box, each pair of the circumferentially adjacent coils share a same circumferential monitoring area (a quarter of the bolt hole perimeter) and distribute separately in the axial direction after the flexible sensing film is winded by adhesive on the bolt surface. Although only one layer of the sensing coil array is plotted in the picture for the sake of brevity, the axial dimension of the sensing array is set as 3 in this paper, which should be decided by the actual demand.

The part highlighted by a red dashed box at the lower part of Figure 2 describes half of the simplified winding model of the adjacent circumferential receiving coils. As has been demonstrated, the induced voltage of one sensing coil will change if and only if the crack reaches and extends in its area. Thus, as a result of the wingding configuration of the triangular coil, the effective coil turns feeling the change of the EC field at a specified circumferential position from the left to right decrease for S1, while increase for S2. For example, when the crack is at the blue line, there are 5 effective turns for S1 and 2 effective turns for S2. It is expected that the induced voltage of the sensing coil will vary linearly with the circumferential position of the crack.

### 2.1. Finite Element Simulation Model

The triangular coil winding on the bolt and the crack on the hole edge make the geometry of the structure more complex. It is difficult to obtain an analytical solution. Thus, finite element method was employed to solve the electromagnetic field equations and calculate magnetic vector potential A and electric scale potential φ

(1) $$\nabla^{2}\varphi -\mu \varepsilon \frac{\partial^{2}\varphi }{\partial t^{2}}=-\frac{\rho }{\varepsilon }$$

(2) $$\nabla^{2}A-\mu \varepsilon \frac{\partial^{2}A}{\partial t^{2}}=-\mu J$$

where ρ denotes free charge bulk density, μ is permeability, ε is dielectric constant, and J is conduction current density.

**Figure 3:**
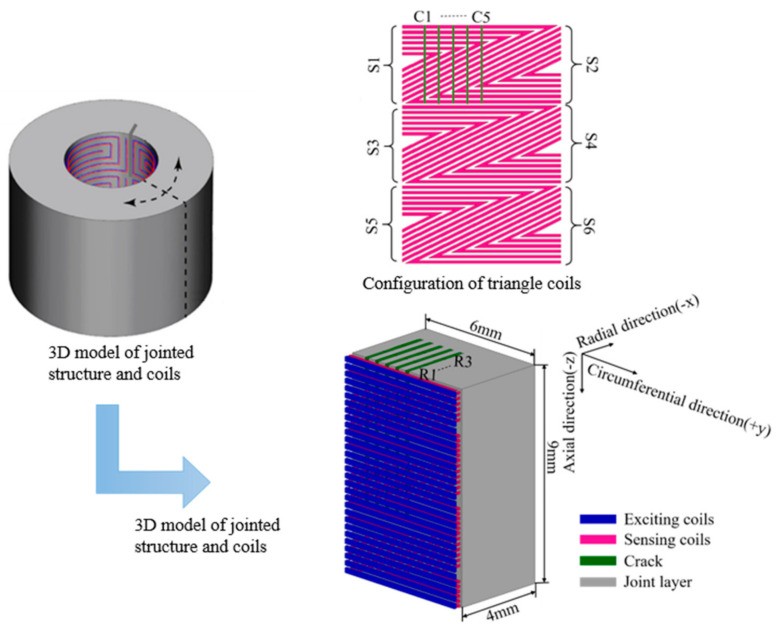
The simulation model.

The finite element simulations are conducted in Ansoft Maxwell. There are two kinds of the exciting coil considered in the paper, the rectangular one in Model 1 and the triangular one in Model 2. All of the parameters other than the exciting coils are same in both models, including the sensing coils, the joint layers and the running parameters. The joint layer with an electrical conductivity of 38 MS/m and a relative permeability of 1.00021 measures 6 mm in length, 4 mm in width and 9 mm in height, shown in Figure 3. To demonstrate the ability of the sensing coils to identify the crack angle and to track the crack growth in the radial direction, crack elements with a dimension of 1 mm × 0.2 mm × 3 mm are adopted to switch the crack position and to give rise to a mesh consistent in each crack state. Five circumferential positions marked from C1 to C5 are set from the left side to the upper middle of the model with a step of 0.55 mm. At each circumferential position, there are 3 crack elements in the radial direction, marked from R1 to R3. When the crack is at CiRj, which means the crack is at the circumferential position Ci and its radial length is jmm, the corresponding crack elements’ material will be set as air.

In order to reduce the computational burden, a quarter model is selected for the simulation according to the fine symmetry in the sensing coils when it is wound on the bolt. The turns distance and width are both 0.1 mm for the triangular coils and are set as 0.1 mm and 0.2 mm, respectively, for the rectangular exciting coils. Sensing coils are denoted as S1 to S6 from up to down. A sinusoidal exciting signal with a frequency of 1 MHz and an amplitude of 2 V is applied in the simulation. The induced voltage of each sensing coil is calculated for crack state analysis.

**Figure 4:**
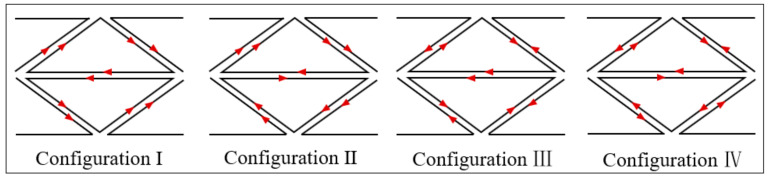
Four exciting configurations.

**Figure 5:**
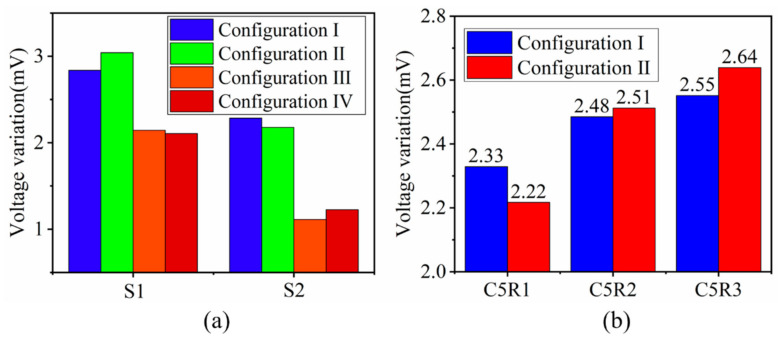
Simulation results for determining the best exciting configuration. (**a**) Voltage variations of the S1 and S2 when the crack is set at C5R1. (**b**) Voltage variations of S2 when the crack propagates in the radial direction at C5.

**Figure 6:**
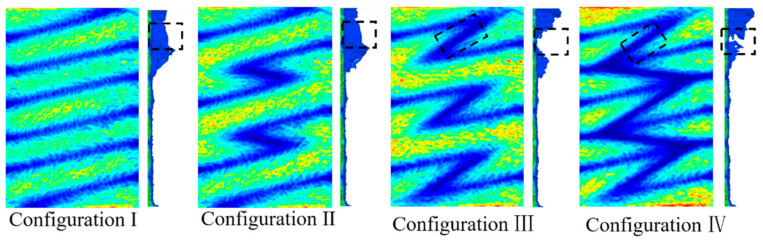
Current density distribution.

The exciting current direction, which has a significant influence on the crack detection, needs to be determined first. Shown in Figure 4, according to the current directions (same or opposite) between the axially/circumferentially adjacent triangular coils, there are 4 configurations compared in the simulation, numbered from configuration I to configuration IV. For example, in configuration II, the current directions of the axially adjacent coils are opposite and those of the circumferentially adjacent coils are same. The best exciting mode is determined by comparing the induced voltage variation of S1 and S2 when a crack is set at C5R1. The simulation results are plotted in Figure 5. It can be found that for both S1 and S2, the induced voltage variation is obviously greater when the circumferentially adjacent coils have same excitation current directions at the adjacent position (Configuration I, Configuration II). To explain the above result, the front view of the EC density when the test specimen is integrated and the side view when there is a crack at C5R1 are displayed in Figure 6 draw by Matlab.

Figure 6 shows that the EC vanishes between the S1 and S2 (highlighted by a dashed box on the side view) in configuration III and configuration IV, which leads to less EC flowing along the radial direction (highlighted by a dashed box on the side view). Therefore, the induced voltages of the S1 and S2 in configuration III, Configuration IV change less than that of the configuration I and Configuration II.

**Figure 7:**
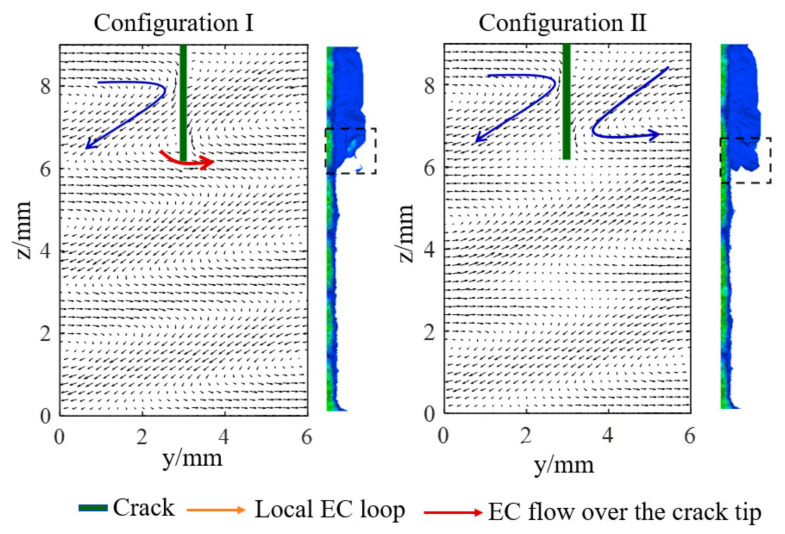
EC vector and cloud picture when the crack is at C5R1.

However, the similar effects of configuration I and configuration III make it impossible to decide which is better to use to detect cracks. To address this problem, additional simulations in which the crack grows from C5R1 to C5R3 are performed to further determine the best exciting mode. The results are shown in Figure 5b where only the voltage variation of S2 is plotted because S2 is more spatially correlated with the exciting current direction between the axially adjacent coils. Although the induced voltage of configuration II changes less at first, it changes greater than that of Configuration I when the radial length of the crack is 2 mm and 3 mm, which indicates that Configuration II has better capability to track crack growth in the radial direction. Likewise, the reason for the phenomenon is given by the combination of the EC cloud picture and the EC vector on the surface of the test specimen when the crack is at C5R3, as shown in Figure 7. From the EC cloud picture, it can be seen that EC flows slightly more in the radial direction, which can be explained as follows. First of all, it is worth stressing that there are 3 modes when EC flows conductively: (1) EC flows over the crack tip when the EC flows in the same direction around the crack tip (red arrow); (2) a local EC loop is built because of the opposite EC direction under two adjacent coils (blue arrow); (3) EC flows in the radial direction, which causes a decrease of EC on the surface of the conductive test specimen (side view). EC always finds the shortest way to flow. Therefore, when the crack tip is at the middle of two adjacent coils with the same exciting direction, it is easier for EC to flow over the crack tip, leading to less EC flowing along the radial direction especially when the radial length of the crack is long enough, which results in less voltage variation in Configuration I when the crack is at C5R2 and C5R3. As for Configuration II, compared to flowing over the crack tip by overcoming the original EC direction, it seems more easier for EC to flow along the radial direction. In conclusion, Configuration II is selected as the final exciting form for Model 2. For Model 1, opposite exciting current directions are applied in axially adjacent coils, and there is no need to consider the current directions in circumferentially adjacent coils because of the space configuration of the rectangular coils.

### 2.2. Simulation Results

#### 2.2.1. Crack Angle Identification

**Figure 8:**
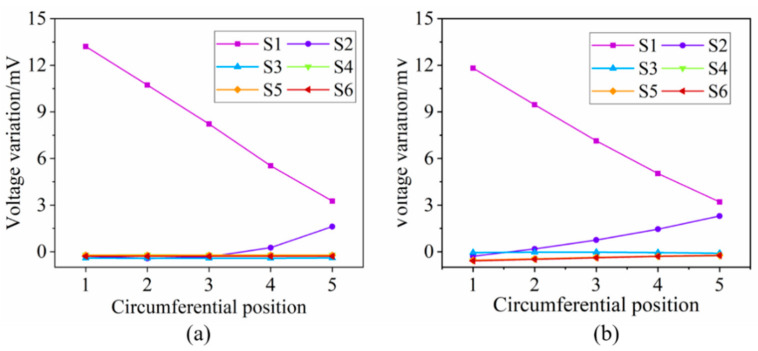
Simulation results of the crack angle detection. (**a**) Model 1 (rectangular exciting coils); (**b**) Model 2 (triangular exciting coils).

**Figure 9:**
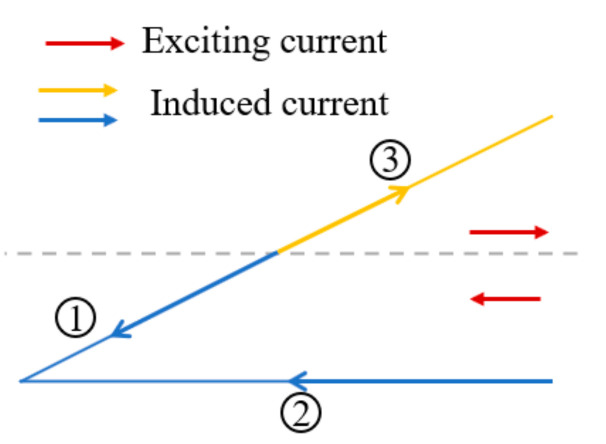
A schematic diagram of the inductive current directions in one triangular turn in Model 1.

**Figure 10:**
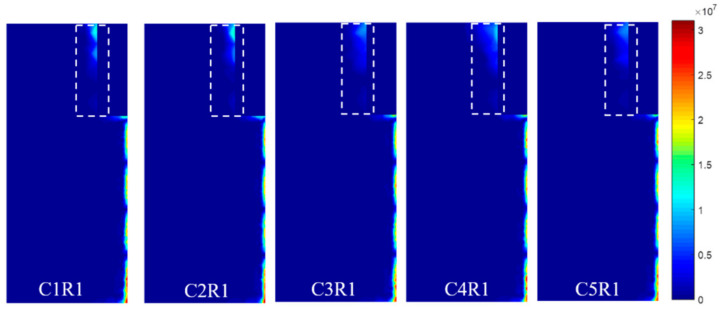
The side view of EC in Model 1.

Figure 8a and Figure 8b present the crack angle identification results for Model 1 and Model 2 respectively. As shown in Figure 8a, the induced voltage variation of S1 decreases linearly and that of the S3–S6 keeps stable when the circumferential position of the crack changes from C1 to C5, which meets the expected design goal. However, compared to Model 2, the inducted voltage of S2 in Model 1 changes little until the crack is at C4 and C5, which indicates that the rectangular exciting coil deteriorates the crack angle identifying ability of the triangular sensing coil. This phenomenon can be explained by Figure 9 and Figure 10. It can be seen from Figure 9 that a single turn can be divided into 3 parts according to the exciting direction bounded by a dashed line. It should be noted that the induced voltage of the whole single-turn coil can be represented by the sum of the 3 parts and the direction of the voltage variation caused by the crack is same for the 3 parts. Assuming the exciting direction and the induced voltage direction at a certain time is as shown in Figure 10, it is obvious that the effects of the part 1 and part 3 offset, while those of part 2 and part 3 should be summed up. As a result, the induced voltage of the S1 changes little when the crack is at C1–C3, though there is much EC flowing in the radial direction shown in the cloud picture in Figure 10, which actually contributes to the increasing induced voltage at a macroscopic level.

**Figure 11:**
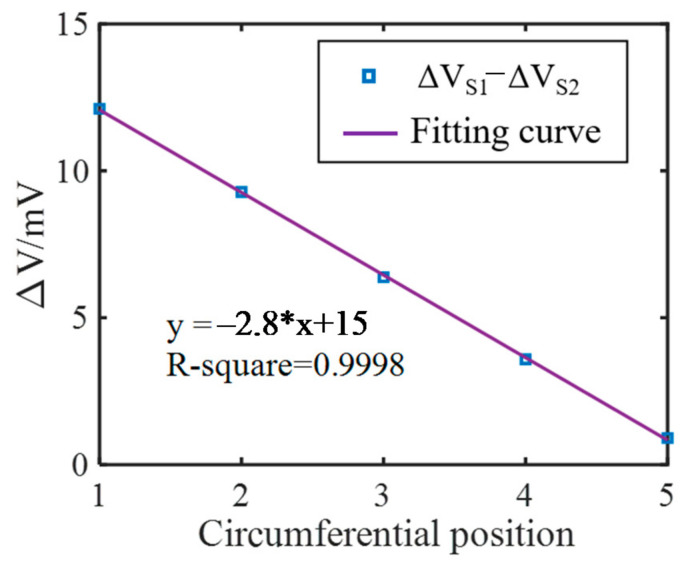
The difference voltage of the S1 and S2 versus the crack circumferential position.

**Figure 12:**
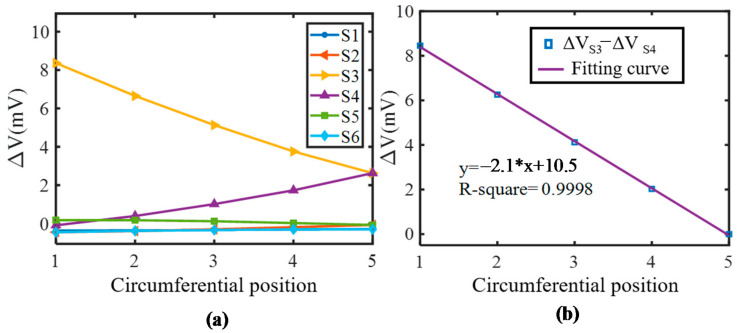
Simulation results for the second layer. (**a**) Voltage variation of each coil; (**b**) Difference value between adjacent coils.

The crack angle position is further represented by the difference of the induced voltage of S1 and S2, as shown in Figure 11. A fine linearity can be found in the fitting curve with a R-square of 0.9998. The simulation model consists of 3 layers in the axial direction. The first layer and the third layer are axially symmetrical. Therefore, simulations when the crack is at the second layer are also performed and the results are displayed in Figure 12. This shows that the results are similar to the first layer.

In conclusion, triangular exciting coils achieve a better ability to identify the crack angle compared to rectangular exciting coils when triangular coils are applied as inductive coils. Meanwhile, triangular coils achieve good linearity with the crack angle, which is a very important property of a sensor.

#### 2.2.2. Tracking Crack Growth in the Radial Direction

**Figure 13:**
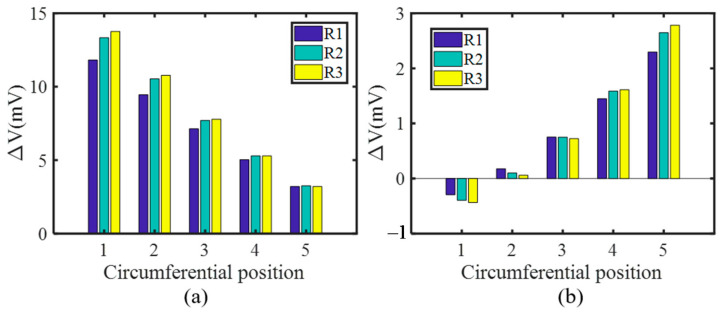
The induced voltage variation of S1 and S2 when the crack propagates along the radial direction at each circumferential position. (**a**) S1; (**b**) S2.

Simulations where the crack propagates along the radial direction are performed as well. Figure 13a and Figure 13b show the voltage variation of S1 and S2 respectively. The following can be concluded from the picture: (1) the induced voltage of S1 increases gradually as the radial length of the crack increases at the circumferential position C1–C4, while that of the S2 increases only at C4 and C5; (2) the ability of the S1 to monitor the crack growth in the radial direction deteriorates as the circumferential position changes from C1 to C5, which can be attributed to the decrease of the effective coil turns; (3) S2 achieves a better effect at C5 than S1, further demonstrating the fact that the opposite exciting current direction in adjacent coils is constructive to track the radial length of the crack. Furthermore, although the voltage variation trends of the S2 shows an inability to track the crack growth at position C1–C3, S1 alone is enough as long as the circumferential position is identified.

#### 2.2.3. Tracking Crack Growth along the Axial Direction

**Figure 14:**
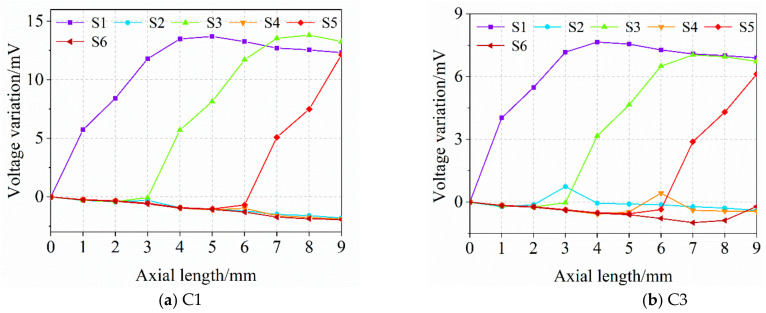
Voltage variation versus axial length.

An early crack that initiates from the hole edge of the joint layer often propagates along the axial direction at first. As a result, several simulations were carried out and the output voltage of each sensing coils were extracted and analyzed to evaluate the applicability of the triangular sensing film to monitor the crack growth in the axial direction. The crack starts from the first layer at two typical circumferential positions, C1 and C3, respectively, and propagates with a step of 1 mm along the axial direction. The results are presented in Figure 14. This shows that the axial length of the crack can be acquired by comparing the induced voltage of all sensing coils based on the following principles: (1) the induced voltage increases compared to the baseline data when the crack is at its monitoring area; (2) the induced voltage of the coil in accordance with principle (1) increases when the crack propagates in its monitoring domain; (3) when the crack has covered its axial length at a certain crack angle, the output voltage of the sensing coils in accordance with (1) and (2) will stay at a stable level, which is enough to decide that the crack exists within its axial length; (4) the space configuration of the sensing coils is settled. Taking 4 mm in Figure 14b as an example, first, it shows in Figure 14b that only the induced voltage of the S1 and S3 greatly increases which means that the axial length of the crack is between 3 mm and 6 mm. The crack angle can be obtained roughly by the nearly unchanged output value of the S2 and further deferred by S2 through the crack angle detection line in Figure 8. Second, the axial range of the crack tip can be further narrowed by the current voltage variation (about 3.2 mV) of the S2 and the maximum one (about 6 mV) when the crack length covered the S3 shown in Figure 12.

## 3. Experiments

**Figure 15:**
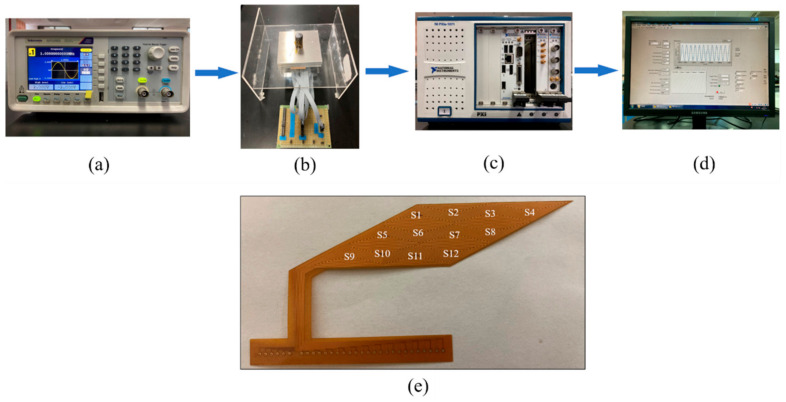
Experimental setup and sensor film. (**a**) Signal generator; (**b**) intelligent bolt; (**c**) signal receiver; (**d**) signal displayer; (**e**) eddy current sensing film.

The performance of the EC sensing film with triangular exciting coil and triangular sensing coil array is evaluated by experiments, and the experimental setup is shown in Figure 15. The exciting signal with an amplitude of 2 V and a frequency of 1 M was generated by a signal generator made by Tektronic. The induced voltage of each coil is acquired through a channel switch and signal receiver made by National Instruments with a sample frequency of 20 M and a sample length of 20,000. Thus, the signal measurement and processing can be automatically finished nearly real-time, which shows a good monitoring efficiency similar to other SHM methods. With regard to the test specimen, three rectangular aluminum (6061) plates, each of which has dimensions 75 mm (length) × 75 mm (width) × 5 mm (thickness) and a bolt hole with diameter 13.5 mm at the center, were jointed by a steel bolt. The triangle eddy current sensing film, with dimensions 40.7 mm × 15 mm × 0.2 mm, was made by a flexible printed circuit process. In the real engineering applications, a wear-resistant coating layer of SiO_2_ and Ta_2_O_3_ can be deposited on the surface of the sensing film via electron beam physical vapor deposition to improve the friction-resistance performance of the sensing film. As shown in Figure 14, there were 12 independent triangle coils as receiving, while similar 12 triangle coils with same shapes were connected with each other as transmitting according to Configuration II in Figure 4.

### 3.1. Crack Angle Identification

**Figure 16:**
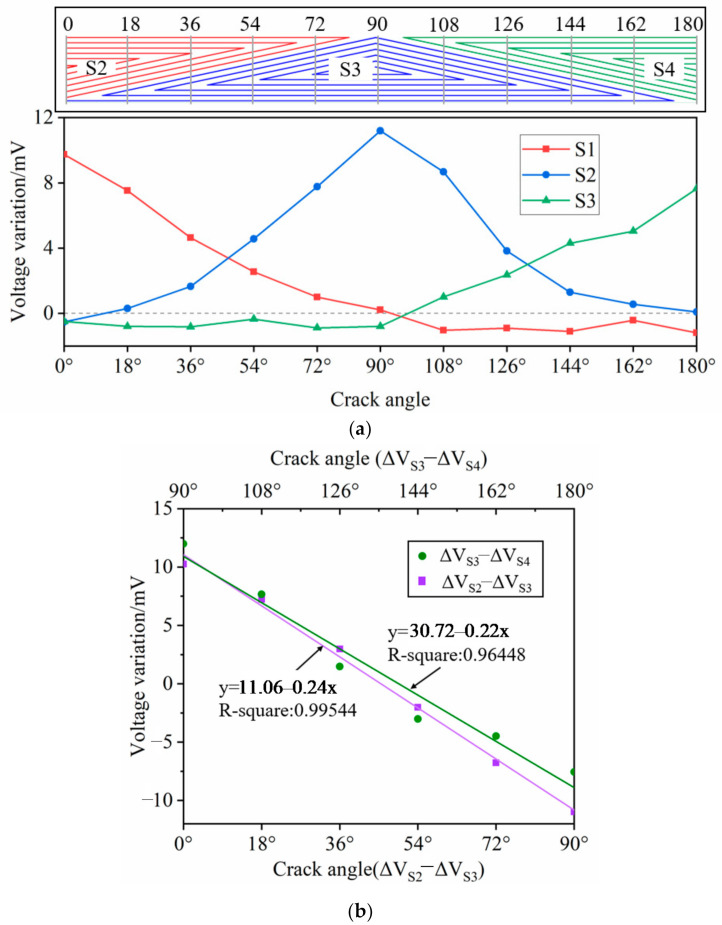
Crack angle detection results. (**a**) Voltage variation of the S2–S4 versus crack angle; (**b**) Voltage difference of S3 (S2) and S4 (S3).

The crack was set at the hole edge of the uppermost plate by spark erosion and the crack angle was changed by rotating the specimen from the middle of the S2 (0°) to the middle of the S4 (180°) with a step of 18°. For the sake of accuracy, the reference voltage under each crack angle was recorded. The positions of the crack represented by gray lines are shown schematically on the top of Figure 16a. As seen in Figure 16a, the voltage variation trends are consistent with the simulation: the output voltage is proportional to the effective sensing turns at a certain circumferential position. The voltage variation of S2 decreases as the crack angle changes from 0° to 90° and keeps stable when the crack is at the position of S3 and S4, while that of the S4 increases when the crack angle changes from the middle of S3 to the middle of S4, which allows the crack angle identification by the sensing film. Furthermore, the voltage difference of the S2 and S3 as well as the S3 and S4 is calculated and plotted in Figure 16b, in which both achieve a good linearity with the crack angle, showing that the proposed triangular sensing film performs well in crack angle identification.

It can be seen from the above results that when there is only a single crack in the circumferential angle monitoring area of a single crack, there is a linear relationship between the circumferential angle of the crack and the induced voltage difference of the receiving coil

(3) ϑ=kΔV+b

where ϑ is the circumferential angle of the crack, k is the slope of a straight line, ΔV is the difference of the induced voltage variation of the relevant receiving coil, and b is the intercept of the straight line.

In the real engineering application, the linear relationship is easy to obtain as the baseline for the crack angle quantification.

### 3.2. Crack Growth in the Radial Direction

**Figure 17:**
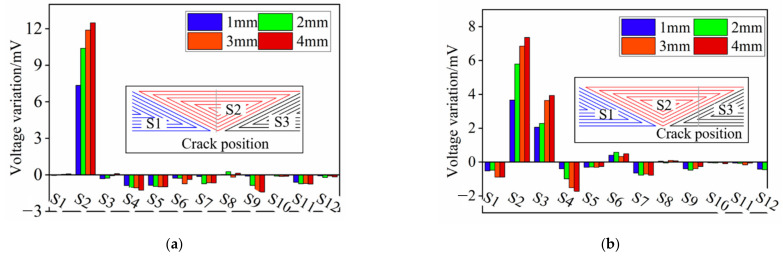
Experimental results when the crack grows in the radial direction at two circumferential positions. (**a**) Voltage variation when the crack position is at the middle of the S2; (**b**) voltage variation when the crack position is in the middle of the S2 and S3.

Two experiments in which two typical circumferential positions were considered were conducted to verify the ability of the proposed sensing film to track crack growth in the radial direction. The crack position was set at the middle of the S2 in the first experiment and was set at the middle of two circumferentially adjacent coils (S2 and S3 in the experiment) in the second experiment. The results are given in Figure 17. First, when the crack was at the middle of the S2, only the output voltage of the S2 increased obviously when the radial length of the crack increased from 1 mm to 4 mm in a step of 1 mm. When the crack was at the middle of the S2 and S3, both the induced voltage of the S2 and S3 increased and that of the S2 increased more than that of the S2, as a result of more effective turns as shown in the crack position schematic in Figure 17b. Another point worth mentioning is that the voltage variation changes in a smaller way with the increase of the radial length of the crack, implying that the radial measuring rage of the film is not very large (but at least 4 mm in this experiment) but enough to be taken seriously in real engineering applications. It may be improved by increasing the exciting voltage amplitude, reducing the exciting frequency or increasing the equipment accuracy. Results for the coil voltage are in excellent agreement with those obtained with the finite-element method.

### 3.3. Crack Depth Identification

**Figure 18:**
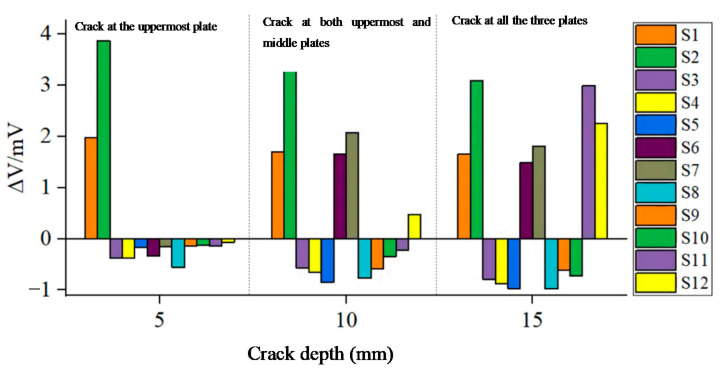
Experimental results as the crack grows in the axial direction.

Three cracks, with angle at the circumferential center of Coils S2 and S1, were set at the hole edge of three jointed plates, respectively. The radial length of the crack was set as 1 mm. The results in Figure 18 show that the crack depth can be inferred by the induced voltage. When one plate has a hole-edge crack, the induced voltage of the coil at the axial location of this plate has an obvious change. For example, when one crack occurs at the location of the uppermost plate, only the induced voltages of S1 and S2 are enhanced significantly. When two cracks are present at the locations of both uppermost and middle plates, only the induced voltages of coils S1, S2, S6 and S7 change considerably, while the induced voltages of other coils change less. This indicates that the proposed sensing film has a good capability of identifying crack depth, at least, identifying which plate has a hole-edge crack for multi-layer bolted joints.

## 4. Conclusions

Enhancing the damage quantification level of bolted joints has been always the research hotspot of aircraft structural health monitoring. In this paper, a novel triangle eddy current sensing film is proposed to quantitatively identify the hole-edge crack of bolted joints. The conclusions are as follows:

(1) The sensing film includes an exciting coil and multiple triangle sensing coils distributed in the axial and circumferential directions of the bolt hole. By analyzing the eddy current distribution and effect of crack quantification, five exciting-coil configurations of the sensing film, including one rectangle-coil configuration and four types of triangle-coil configuration, have been compared to give the best configuration of the eddy current sensing array.

(2) There is an approximate linear relationship between the crack angle and the difference of the induced voltage variation between two adjacent triangle sensing coils in the same layer, which can be used to quantify the crack angle with a good accuracy.

(3) By simulation and experimental analysis, the proposed sensing film has a good capacity not only to identify the crack angle, but also to quantify the crack length in the radial direction and the crack depth in the axial direction of the bolt hole.

However, some further studies will be conducted in the future for real engineering applications of this sensing technology:

(1) The configuration of the sensing film or the measuring parameters of the system should be optimized to improve the measuring range of the crack radial dimension.

(2) The robustness of the sensing film should be further verified in real engineering applications, which may include the influence of environmental variation and bolt looseness.

(3) In this paper, forward analysis between the induced voltage of eddy current coils and the crack parameters has been conducted to show the capacity of the proposed sensing film. The backward problem that the proposed sensing film is used to detect the crack will be further studied and verified in future.

## References

[1] Najafi A., Garg M., Abdi F. Failure analysis of composite bolted joints intension. Proceedings of the 50th AIAA/ASME/ASCE/AHS/ASC Structures, Structural Dynamics, and Materials Conference.

[2] Oskouei R.H., Keikhosravy M., Soutis C. (2016). A finite element stress analysis of aircraft bolted joints loaded in tension. Aeronaut. J..

[3] Sun H.-T., Chang F.-K., Qing X. (2002). The Response of Composite Joints with Bolt-Clamping Loads, Part I: Model Development. J. Compos. Mater..

[4] Qing X., Li W., Wang Y., Sun H. (2019). Piezoelectric Transducer-Based Structural Health Monitoring for Aircraft Applications. Sensors.

[5] Huo L., Wang F., Li H., Song G. (2017). A fractal contact theory based model for bolted connection looseness monitoring using piezoceramic transducers. Smart Mater. Struct..

[6] Qiu L., Yuan S., Chang F.-K., Bao Q., Mei H. (2014). On-line updating Gaussian mixture model for aircraft wing spar damage evaluation under time-varying boundary condition. Smart Mater. Struct..

[7] Kim Y., Lim H.J., Sohn H. (2018). Nonlinear ultrasonic modulation based failure warning for aluminum plates subject to fatigue loading. Int. J. Fatigue.

[8] Jung H.K., Jo H., Park G., Mascarenas D.L., Farrar C.R. (2014). Relative baseline features for impedance-based structural health monitoring. J. Intell. Mater. Syst. Struct..

[9] Huo L., Chen D., Liang Y., Li H., Feng X., Song G. (2017). Impedance based bolt pre-load monitoring using piezoceramic smart washer. Smart Mater. Struct..

[10] Roach D. (2009). Real time crack detection using mountable comparative vacuum monitoring sensors. Smart Struct. Syst..

[11] Argatov I., Butcher E.A. (2011). On the separation of internal and boundary damage in slender bars using longitudinal vibration frequencies and equivalent linearization of damaged bolted joint response. J. Sound Vib..

[12] Hoang T.D., Herbelot C., Imad A. (2010). Rupture and damage mechanism analysis of a bolted assembly using coupling techniques between A.E. and D.I.C. Eng. Struct..

[13] Goldfine N., Zilberstein V., Washabaugh A., Schlicker D., Shay I., Grundy D. (2003). Eddy current sensor networks for aircraft fatigue monitoring. Mater. Eval..

[14] Zilberstein V., Schlicker D., Walrath K., Weiss V., Goldfine N. (2001). MWM eddy current sensors for monitoring of crack initiation and growth during fatigue tests and in service. Int. J. Fatigue.

[15] Zilberstein V., Grundy D., Weiss V., Goldfine N., Abramovici E., Newman J., Yentzer T. (2005). Early detection and monitoring of fatigue in high strength steels with MWM-Arrays. Int. J. Fatigue.

[16] Sheiretov Y., Grundy D., Zilberstein V., Goldfine N., Maley S. (2009). MWM-Array Sensors for In Situ Monitoring of High-Temperature Components in Power Plants. IEEE Sens. J..

[17] Goldfine N., Zilberstein V., Cargill J.S., Schlicker D., Shay I., Washabaugh A., Tsukernik V., Grundy D., Windoloski M. (2002). Meandering winding magnetometer array eddy current sensors for detection of cracks in regions with fretting damage. Mater. Eval..

[18] Li P., Cheng L., He Y., Jiao S., Du J., Ding H., Gao J. (2016). Sensitivity boost of rosette eddy current array sensor for quantitative monitoring crack. Sens. Actuators A Phys..

[19] Li P.Y., Cheng L., He Y.T., Du J.Q., Jiao S.B., Gao J.Y. (2018). Mutual interference effect of a rosette eddy current array sensor for quantitative crack monitoring in metallic structures. Insight.

[20] Chen G., Zhang W., Zhang Z., Jin X., Pang W. (2018). A new rosette-like eddy current array sensor with high sensitivity for fatigue defect around bolt hole in SHM. NDT E Int..

[21] Fan X., Chen T., He Y., Du J., Ma B., Song Y. (2019). An excitation coil layout method for improving the sensitivity of a rosette flexible eddy current array sensor. Smart Mater. Struct..

[22] He Y., Chen T., Du J., Ding H., Jiao S., Li P. (2017). Temperature-compensated rosette eddy current array sensor (TC-RECA) using a novel temperature compensation method for quantitative monitoring crack in aluminum alloys. Smart Mater. Struct..

[23] Chen T., Du J., He Y., Du J. (2018). A structural crack monitoring gasket for aircraft bolt-jointed structures with temperature compensation. Smart Mater. Struct..

[24] Rakow A., Chang F.-K. (2011). A structural health monitoring fastener for tracking fatigue crack growth in bolted metallic joints. Struct. Health Monit..

[25] Sun H., Wang T., Liu Q., Qing X. (2018). A novel eddy current array sensing film for quantitatively monitoring hole-edge crack growth in bolted joints. Smart Mater. Struct..

[26] Sun H., Wang T., Liu Q., Wang Y., Qing X. (2019). A two-dimensional eddy current array–based sensing film for estimating failure modes and tracking damage growth of bolted joints. Struct. Health Monit..

[27] Liu Q., Sun H., Chai Y., Zhu J., Wang T., Qing X. (2020). On-site monitoring of bearing failure in composite bolted joints using built-in eddy current sensing film. J. Compos. Mater..

